# Interleukin-2 (IL-2) Interacts With IL-2 Receptor Beta (IL-2Rβ): Its Potential to Enhance the Proliferation of CD4+ T Lymphocytes in Flounder (*Paralichthys olivaceus*)

**DOI:** 10.3389/fimmu.2020.531785

**Published:** 2020-09-09

**Authors:** Xiujuan Zhou, Jing Xing, Xiaoqian Tang, Xiuzhen Sheng, Heng Chi, Wenbin Zhan

**Affiliations:** ^1^Laboratory of Pathology and Immunology of Aquatic Animals, KLMME, Ocean University of China, Qingdao, China; ^2^Laboratory for Marine Fisheries Science and Food Production Processes, Qingdao National Laboratory for Marine Science and Technology, Qingdao, China

**Keywords:** IL-2 receptor beta, Interleukin-2, CD4+ T lymphocyte, proliferation, Th1-mediated immune response, *Paralichthys olivaceus*

## Abstract

Interleukin-2 (IL-2) is an important immunomodulatory cytokine that primarily promotes the activation, proliferation, and differentiation of CD4+ T helper subsets and CD4+ T regulatory cells. In our previous studies, IL-2 and IL-2 receptor beta (IL-2Rβ) genes of flounder (*Paralichthys olivaceus*) were cloned, and IL-2Rβ molecules expressed on both B and T lymphocytes were identified. In the present study, the interaction of flounder IL-2 (fIL-2) with the IL-2 receptor beta (fIL-2Rβ) was investigated. The proportion of CD4+ T lymphocytes and IL-2Rβ+ cells were detected both *in vivo* and *in vitro*. Firstly, the binding of recombinant flounder IL-2 protein (rfIL-2) and rfIL-2Rβ was verified by pull-down assay and enzyme-linked immunosorbent assay. Indirect immunofluorescence assay showed that rfIL-2 enhanced the proliferation of CD4+ and IL-2Rβ+ cells in the gill and spleen. Furthermore, CD4-1+, CD4-2+ T lymphocytes and IL-2Rβ+ cells were significantly upregulated in cultured peripheral blood lymphocytes (PBLs) with addition of rfIL-2, as shown by Flow cytometry. The related genes were examined by Q-PCR in cultured PBLs with added rfIL-2. The results showed that the IL-2–IL-2R interaction induced upregulated expression of T lymphocyte surface makers, Th1-related cytokines or transcription factors, and critical genes of the IL-2 signaling pathway. In addition, these IL-2–elicited biological functions and immune responses were downregulated by blocked with anti–rfIL-2Rβ and anti–rfIL-2 Abs, showing that IL-2Rβ plays an indispensable role in IL-2 elicited biological function. Our results demonstrated that the interaction between IL-2 and IL-2Rβ showed its potential to enhance the proliferation of CD4+ T lymphocytes in flounder. As found in mammals, a Th1-mediated mechanism regulated by this interaction exists in teleost.

## Introduction

In mammals, Interleukin-2 (IL-2), a four α-helical bundle cytokine with a molecular weight of 15kDa, is a T cell growth factor that is mainly produced by activated T cells (1). Further, IL-2 is a critical Th1-type cytokine that plays an important role in immune regulation, wherein it primarily promotes the activation, proliferation, and differentiation of T cells, B cells, and NK cells (2, 3). Activated T cells, including CD4+ and CD8+ T cells, secrete a high amount of IL-2 to induce autocrine effects as well as paracrine stimulation of their neighboring IL-2 receptor (IL-2R)-expressing cells (4, 5).

IL-2 exerts biological functions by specifically binding with its receptor, which consists of three subunits, namely IL-2Rα (CD25), IL-2Rβ (CD122), and γc (CD132); both the IL-2Rβ and γc chains belong to a type I cytokine receptor superfamily and are responsible for signaling (6, 7). IL-2 binds to the receptor complex to elicit intracellular signaling events that ultimately lead to cell proliferation (8, 9). IL-2 binding to IL-2R to form a ligand-receptor complex triggers the phosphorylation of transcription factors, leading to induction of gene expression. The resulting signal transduction occurs via three major pathways, JAK-STAT, PI3K-AKT, and MAPK (10).

IL-2 activity has been demonstrated to be present in supernatants of activated T cells, and is able to mediate human T cell growth and proliferation (11–13). In addition, IL-2 has been shown to promote the differentiation of naïve CD4+ T cells into T helper 1 (Th1) and T helper 2 (Th2) cells, while inhibiting differentiation into T helper 17 (Th17) and T follicular helper (Tfh) cells (14). Thus, IL-2 has broad essential biological activities in driving T cell proliferation and modulating effector cell differentiation. As a crucial immune cell, T lymphocytes participate in immune response of fish. Our previous study showed that IL-2Rβ is expressed on CD4+ T lymphocytes (15). However, to date, direct evidence for the regulation and effect of IL-2 on CD4-1/CD4-2+ T lymphocytes of fish remains to be intensively analyzed. In the current study, the recombinant protein His-IL-2 and GST-IL-2Rβ of flounder (*Paralichthys olivaceus*) were produced successfully. T lymphocyte was analyzed after rfIL-2 addition and injection experiment. Furthermore, anti-rfIL-2Rβ and anti–rfIL-2 antibodies blocking assay on rIL-2 function was examined, respectively. Additionally, the expression of IL-2 signaling pathway genes was investigated. The results provide functional insights into the existence of a conserved Th1-like regulatory mechanism in teleost, enriching our knowledge of fish immunology and contributing to improved understanding of the biological functions of IL-2 and IL-2R as well as the Th1-mediated immune response in fish.

## Materials and Methods

### Fish

Flounder (*Paralichthys olivaceus*, 35 ± 5 g and 1.0 ± 0.1 kg) were obtained from an aquafarm in Rizhao, Shandong province of China, acclimated in the wet lab, and were kept in a circulating water bath with commercial pellets (Great Seven, Qingdao, China) at a daily ration of 0.7% of their body weight. All fish were held in the wet lab for two weeks before performing experiments for acclimatization and evaluation of overall fish health. Only healthy fish, as determined by their general appearance and level of activity, were used in the following experiments. Flounder weighing 1.0 ± 0.1 kg were used for leukocyte preparation and Cell stimulation experiments *in vitro*. Flounder weighing 35 ± 5 g were used for injection experiments.

### Antibodies

Rabbit anti-flounder CD4-1/CD4-2/IL-2/IL-2Rβ and mouse anti-flounder IL-2 (fIL-2) polyclonal antibodies were produced previously in our laboratory (15–17). In this study, rabbit anti-flounder CD4-1/CD4-2 polyclonal antibodies (diluted 1:1000) were used for flow cytometry (FCM) and indirect immunofluorescence assay. Rabbit anti-fIL-2-2Rβ and mouse anti-fIL-2-2 polyclonal antibodies (diluted 1:500) were used in blocking experiments.

### Expression and Purification of His-rfIL-2 and GST-rfIL-2Rβ

The recombinant proteins were expressed and produced as described previously (18). Sequences encoding IL-2Rβ(rfIL-2Rβ) were amplified by PCR using primers (**Supplementary Table S1**) containing an *Eco*RI or *Sal*I site added on the 3’end. The resulting rfIL-2Rβ amplified production was digested and ligated into pGEX-4T-1 (Invitrogen Life Technologies). Plasmid DNAs were transformed into BL21 cells (DE3; Invitrogen Life Technologies). A single BL21 colony harboring the expression plasmids was inoculated into 100 ml of Luria-Bertani medium containing ampicillin (25 mg/l), and the culture was shaken and incubated at 37°C until the OD_600_ value was 0.6. And then *Escherichia coli* containing recombinant protein were induced during exponential growth with isopropylb-D-thiogalactopyranoside (IPTG) for 6 h at 30°C prior to harvesting. After ultrasonication, the soluble supernatant liquids were collected. The recombinant protein was affinity-purified using GSTrap^TM^ 4B (GE Healthcare) according to the manufacturer’s instructions. The recombinant protein IL-2 was produced as described in our previous study (19). The proteins were analyzed by SDS-PAGE and visualized after staining with Coomassie brilliant blue R-250. The concentrations of recombinant proteins were determined using the Bradford method. The recombinant proteins were aliquoted and stored at −80°C.

### GST Pull-Down Assay

For the pull-down binding assays, purified GST-rfIL-2Rβ protein (GST tag protein alone as the negative control) was mixed with BeaverBeads^TM^ GSH beads (Beaverbio, China) and incubated for 6 h at 4°C. The beads then were washed with GST binding buffer (140 mM NaCl, 2.7 mM KCl, 10 mM Na_2_HPO_4_, 1.8 mM KH_2_PO_4_, and 1 mM EDTA, pH 7.4) three times, and washed again with PBS three times by centrifuging at 1000 × *g* for 5 min. Next, His-rfIL-2 diluted in 1 ml of GST pull-down binding buffer was added to the beads collected in the previous step, and incubation was carried out on a horizontal rotator at room temperature overnight. The binding beads were collected by centrifugation at 1000 × *g* for 5 min, and washed three times by centrifugation using GST pull-down binding buffer. After extensive washing, the retained proteins were analyzed by 12% SDS-PAGE.

### Enzyme-Linked Immunosorbent Assay

Direct binding enzyme-linked immunosorbent assay (ELISA) was used to verify the interaction of His-rfIL-2 with GST-rfIL-2Rβ. Between each successive step, the plats were washed three times with PBST. Briefly, recombinant GST-rfIL-2Rβ was coated on a 96-well plate overnight at 4°C. After being blocked with 3% BSA for 1 h at 37°C and washed with PBST, the wells were incubated with His-rfIL-2 protein diluted to different doses (0, 31.25, 62.5, 125, 250, 500, and 1000 μg/ml) at 37°C. After 10 h of incubation at room temperature, the plates were washed and incubated with anti-His tag (1:4000, Sigma) for 1.5 h at 37°C. After the plates were washed again, goat-anti-mouse Ig-alkaline phosphatase conjugate (GAM-AP, 1:5000, Sigma) was added. After washing three times, 100 ml 0.1% (w/v) p-nitrophenyl phosphate (pNPP, Sigma) in 50 mM carbonate-bicarbonate buffer (pH 9.8) containing 0.5 Mm MgCl_2_ was added to each well and incubated for 30 min at room temperature in the dark. The reaction was stopped with 50 μl per well of 2M NaOH, and absorbance was measured with an automatic ELISA reader at 405 nm. The rGST (GST tag protein) as a control, which was replaced GST-IL-2Rβ to react with rIL-2. Similarly, recombinant flounder IL-6 as a control (20), which was replaced rIL-2 to react with GST-IL-2Rβ, with the same procedure. Each sample was assayed three times.

### EdU Injection

The injection experiment was performed as described by Xing et al. (21), with slight modification. In brief, heathy flounder were randomly divided into three groups (15 fish per group). On the day of immunization, PBS, PHA (1 mg/mL) + His-tag protein (1 mg/ml), and PHA (1 mg/ml) + rfIL-2 (1 mg/ml) were injected into fish intraperitoneally (100 μl per individual). After immunization 24 h, EdU (EdU powder dissolve with PBS, 200 μg per individual, RiboBio Co., China) was injected fish intraperitoneally. Then, the spleens and gills were collected from six fish in each group at 7 day post immunization to injection or administration, and used for cryostat sections preparation.

### Cryostat Sections and Indirect Immunofluorescence Assay

The spleen and gill were treated for preparation of cryostat and ultrathin sections by immersing in tissue-freezing medium (OCT, JUNG) immediately and freezing at −80°C; thereafter, 5-μm-thick sections were cut and fixed with cold acetone for 10 min, and stored at −20°C prior to use after air-drying in a fume cupboard (22).

To analyze the effect of rfIL-2 on CD4+ and IL-2Rβ+ cells, spleen and gill cryostat sections were utilized for CD4 or IL-2Rβ and EdU staining. In brief, after washing in PBST (PBS containing 0.5% Tween 20) for 10 min, sections were incubated with EdU solution (RiboBio Co., China) at room temperature in the dark for 30 min. Then, sections were washed with PBST three times, for 5 min each time. Next, the sections were incubated with mixed rabbit anti-CD4-1/CD4-2 or anti-IL-2Rβ antibodies, respectively, and Alexa Fluor 649-conjugated goat anti-rabbit IgG (1:1000 diluted in PBS, Sigma) were used as secondary antibodies. For immunofluorescence observation under a microscope, cryostat sections were counterstained with DAPI (Bio-Legend) for 10 min at 37°C in the dark. Unimmunized rabbit serum was used as primary antibody for negative controls. Then, visualization was performed under a fluorescence microscope (Evos FL Auto2, Thermo, United States).

The quantitative image analysis for co-localization was performed using the image analysis software Image J, a public domain Java image processing program. In the inverted image, green and magenta are reversed, red and aqua are reversed, and blue and yellow are reversed. And then, fluorescence correction of image. The fluorescence density value of each imaging point in the selected area were calculated. The proportion of monochromatic fluorescence and co-localization fluorescence can be calculated and the pie chart will be drawn.

### Cell Stimulation

Leukocytes were isolated from flounder peripheral blood lymphocytes (PBLs) using discontinuous Percoll (Pharmacia) gradient (1.020/1.070), according to the method described in our previous study, with aseptic technique (23). Then, PBLs were transferred to 24-well culture plates (800 μl/well) to ensure 5 × 10^6^ cells per well, and cultured in L-15 medium with 10% fetal calf serum and penicillin/streptomycin supplemented with PBS, PHA (5 μg/well) + His-tag protein (100 ng/well), and PHA(5 μg/well) + rfIL-2(100 ng/well), for 72 h at 22°C. After 72 h stimulation, PBLs were collected for FCM and Q-PCR analysis. The cell survival rate of isolated PBLs was 98.8%, and that of PBLs cultured for 72 h was 84.1% by PI-dye (Thermo Fisher Scientific, catalog number: 00-6990, data not shown), the cell state is suitable for further experiment.

To further verify the effect of IL-2 and IL-2Rβ on lymphocytes, blockade assays were performed. PBLs were cultured in 24-well plates at 22°C. Anti-rfIL-2Rβ antibody were diluted with L-15 medium (at a concentration of 1 μg/ml) and incubated with PBLs at 22°C for 2 h. After the supernatant was removed, PHA(5 μg/well) + rfIL-2(100 ng/well) was added to the cells, as described above. But in anti-rfIL-2 blocked experiment, the protocols were slightly adjusted. Specifically, the anti-rfIL-2 antibody was diluted with L-15 medium (at a concentration of 1 μg/ml), and then mixed with PHA(5 μg/well) + rfIL-2(100 ng/well) was incubated with PBLs at 22°C for 72 h. In parallel, the same concentration of non-specific rabbit/mouse IgG was used as negative control. After 72 h of stimulation, the leukocytes were collected for FCM and Q-PCR analysis.

### Flow Cytometric Analysis

Flow cytometric analysis was performed as described in our previous study, with slight modification (24). Briefly, cells stimulated *in vitro* were collected and stained with rabbit anti-CD4-1, anti-CD4-2, or anti-IL-2Rβ antibodies for 1.5 h at 37°C; then, samples were washed three times with PBS containing 5% (v/v) Newborn Calf Serum. Next, they were incubated with FITC-conjugated goat-anti-rabbit IgG (1:1000 diluted in PBS, Sigma) or goat-anti-rabbit Ig-Alexa Fluor^®^ 647 (1:1000, Thermo Fisher Scientific, United States) for 1 h in the dark at 37°C, and then washed again. Unimmunized serum was instead of Abs as primary antibody used as negative control. Then, cell suspensions were analyzed with an Accuri C6 cytometer (BD Accuri, United States).

### Quantitative PCR

For real-time quantitative PCR (Q-PCR), cells stimulated *in vitro* at 72 h were collected and total RNA was extracted with the Trizol method (25). Then, cDNA was synthesized using the PrimeScript^TM^ reagent Kit with gDNA Eraser (TaKaRa, China) according to the manufacturer’s instructions. Next, T cell markers (CD3, CD4-1, CD4-2, and CD8β), related cytokines (IL-2, IL-2Rβ, TNFα, IFNγ, IL-12p40, and IL-10), transcription factors (T-bet, GATA3), and IL-2 pathway genes (AKT, JAK1, PI3K, and SHC1) were quantified using Q-PCR. cDNA was used as template, and Q-PCR was carried out using SYBR Green I Master (Roche, Switzerland) in a LightCycler^®^ 480 II Real Time System (Roche, United States). The specific primers used are shown in **Supplementary Table S1**. cDNA concentrations were adjusted to 100 ng/ml with NanoDrop 8000. The thermal cycling profile consisted of an initial denaturation step at 95°C for 30 s, followed by 45 cycles of denaturation at 95°C for 5s, and extension at 60°C for 30 s. An additional temperature ramping step was utilized to produce melting curves of the reaction from 65 to 95°C. The expression level of genes in blank control individuals was defined as 1. Each assay was performed in triplicate using the 18S gene as an internal control. All data were analyzed using the 2^–ΔΔCt^ method.

### Statistical Analysis

The data are presented as means ± SD. Statistical analysis comprised one-way analysis of variance (ANOVA) and Duncan’s multiple comparisons, performed using SPSS 20.0 software. The level of significance was defined at *p* < 0.05.

## Results

### Production of Recombinant His-rfIL-2 and GST-rfIL-2Rβ Proteins

**FIGURE 1:**
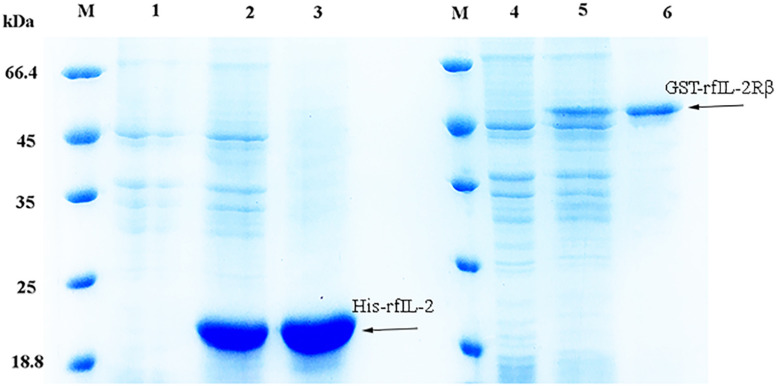
SDS-PAGE of recombinant His-rfIL-2 and GST-rfIL-2Rβ proteins. Lane (L) M: molecular mass marker; L1. whole-cell lysate of non-induced *E. coli*; L2. whole-cell lysate of induced *E. coli* containing recombinant protein of His-rfIL-2; L3. purified recombinant protein His-rfIL-2; L4. whole-cell lysate of non-induced *E. coli*; L5. whole-cell lysate of induced *E. coli* containing recombinant GST-rfIL-2Rβ protein; L6. purified recombinant GST-rfIL-2Rβ protein.

The expression of the recombinant proteins is shown in Figure 1; His-rfIL-2 and GST-rfIL-2Rβ were successfully produced. SDS-PAGE revealed that His-rfIL-2 and GST-rfIL-2Rβ had molecular masses of 20.8 and 49 kDa, respectively, which were consistent with their theoretical molecular masses.

### Binding Assay Between His-rfIL-2 and GST-rfIL-2Rβ

**FIGURE 2:**
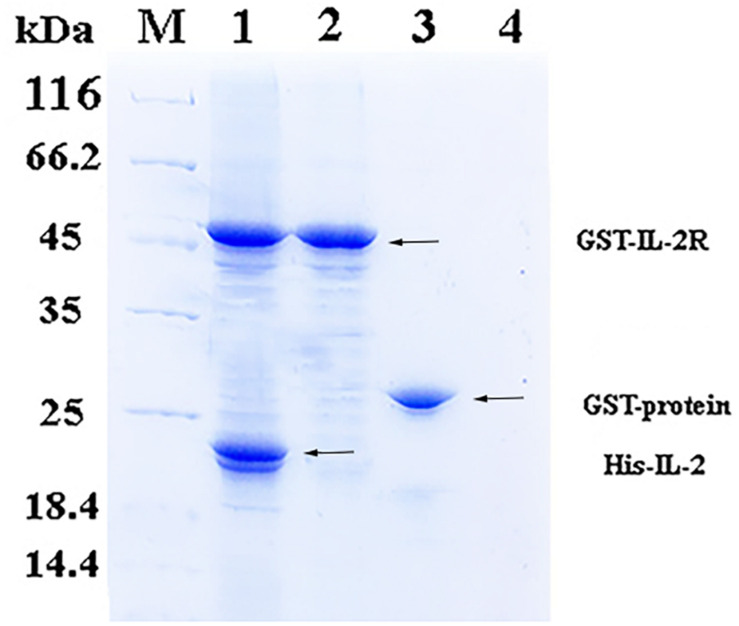
SDS-PAGE confirmed binding between His-rfIL-2 and GST-rfIL-2Rβ by pull-down assay. Lane M, Molecular mass marker; Lane 1, protein profile of BeaverBeads^TM^ GSH beads coupled with purified GST-rIL-2Rβ and His-rIL-2. Lane 2, protein profile of BeaverBeads^TM^ GSH beads coupled with purified GST-rIL-2Rβ and PBS. Lane 3, BeaverBeads^TM^ GSH beads coupled with purified GST-labeled protein and His-rfIL-2. Lane 4, BeaverBeads^TM^ GSH beads coupled with PBS and His-rfIL-2 as negative control.

In the pull-down assay, SDS-PAGE showed two clear bands corresponding to 49 and 20.8 kDa (Figure 2, Line1), which were consistent with the theoretical molecular weights of GST-rfIL-2Rβ and His-rfIL-2. The other lines only showed one band, or no band, in the control groups (Figure 2, Line 2–4). The results revealed that GST-rfIL-2Rβ could efficiently binding with His-rfIL-2.

**FIGURE 3:**
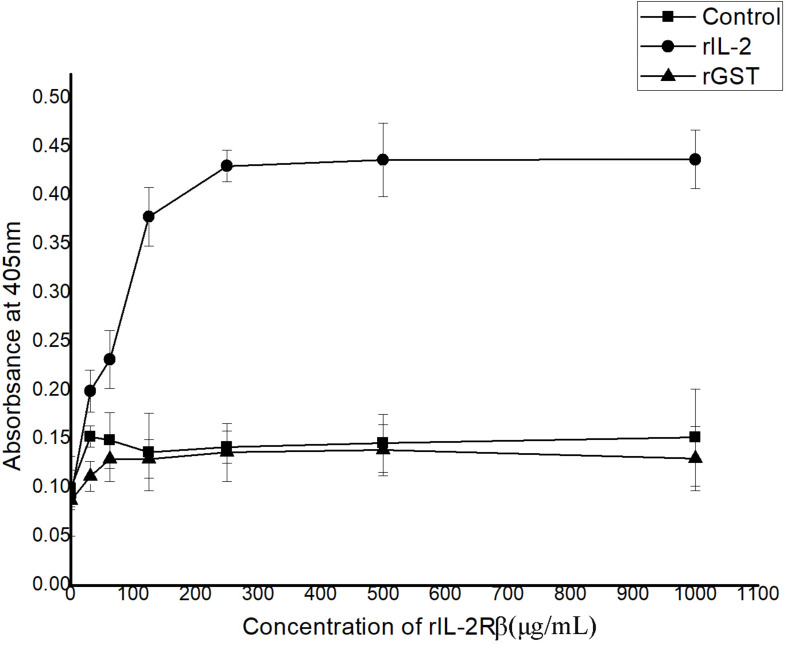
ELISA analysis for the detection of the interaction between purified recombinant GST-rIL-2Rβ and His-rIL-2. The rGST (GST tag protein) as a control, which was replaced GST-IL-2Rβ to reacted with rIL-2. And recombinant flounder IL-6 as a control, which was replaced rIL-2 to reacted with GST-IL-2Rβ, with the same procedure. All data represent results obtained from at least three independent experiments.

In ELISA, interactions between recombinant His-rfIL-2 and GST-rfIL-2Rβ could be detected with the rise in His-rfIL-2 protein concentration at 0–250 μg/ml; when the concentration was more than 250 μg/ml, the OD value tended to be stable. The results indicated that the specific interaction between His-rfIL-2 and GST-rfIL-2Rβ was dose-dependent (Figure 3). However, in the control groups, GST-rfIL-2R**β** failed to bind to recombinant flounder IL-6, and rGST failed to bind to His-rfIL-2, no specific binding was detected in the control group. These results showed that rfIL-2 specifically binds to rfIL-2Rβ.

### RfIL-2 Induced the Proliferation of CD4+ and IL-2Rβ+ Cells in the Gill and Spleen

**FIGURE 4:**
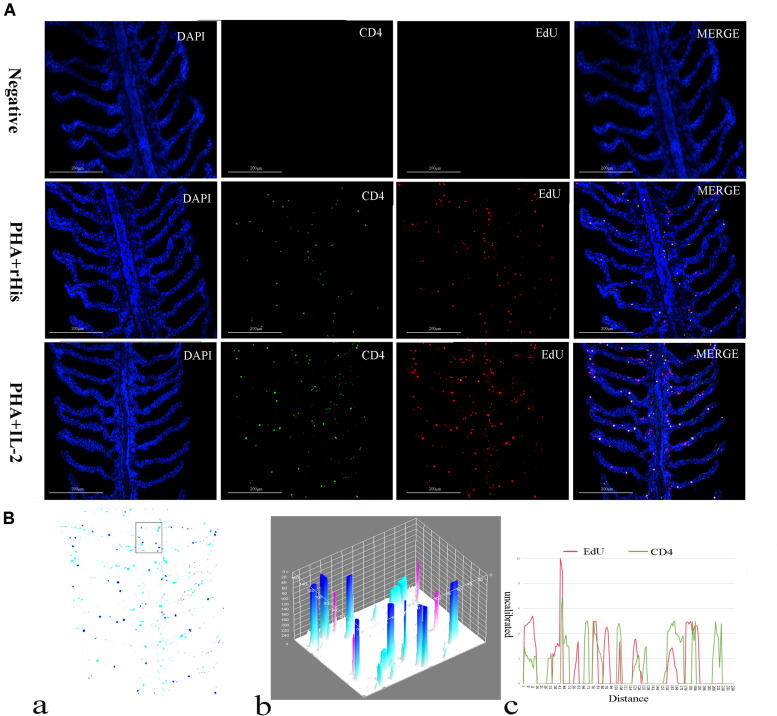
Co-localization analysis of EdU and CD4 in the gill of flounder. **(A)** Indirect immunofluorescence of EdU and CD4 in flounder gill after PHA + rHis, PHA + rHis-IL-2 injection at day 7; bar = 200μm. The cell nuclei were counterstained in blue with DAPI. The unimmunized serum was used as primary antibody for negative control. **(B)** Image analysis for co-localization of EdU and CD4 in a selected area of gill using Image J software. (a) A new merged image showing the PHA + rHis-IL-2 group in **(A)**. Compared with the above image, magenta was represented CD4-positive signals, aqua was represented EdU-positive signals, blue was represented EdU and CD4 double-positive signals, respectively. (b) The interactive 3D surface plot of the selected area (the position of the rectangle) of panel (a). (c) Analysis of co-localization of EdU and CD4 according to the optical density of three fluorescence in the selected area [the position of the rectangle of (a)]. Fifteen fish per group.

**FIGURE 5:**
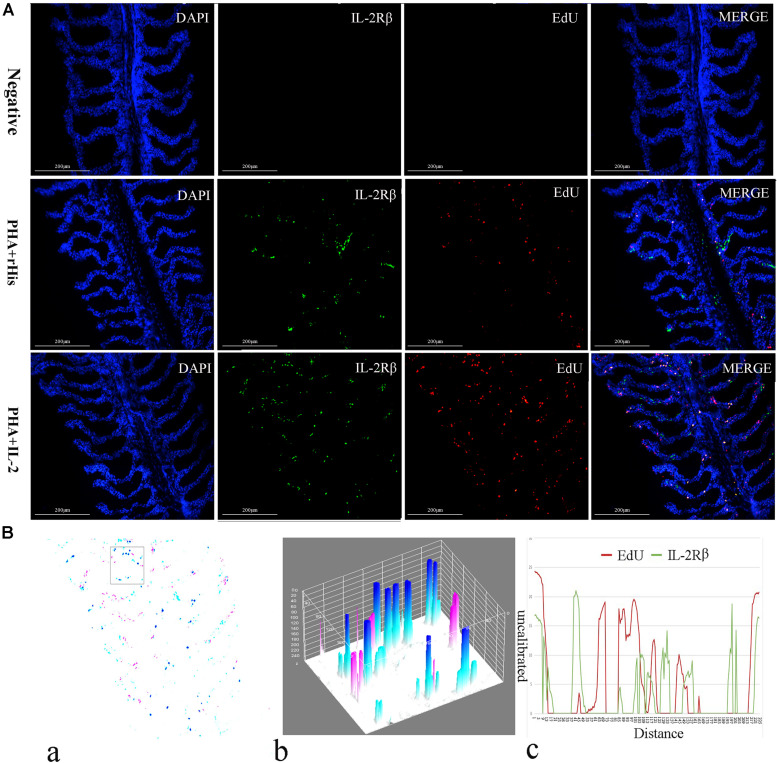
Co-localization analysis of EdU and IL-2Rβ in flounder gill. **(A)** Indirect immunofluorescence of EdU and IL-2Rβ in flounder gill after PHA + rHis, PHA + rHis-IL-2 injection on day 7; bar = 200μm. The cell nuclei were counterstained in blue with DAPI. The unimmunized serum was used as primary antibody for negative control. **(B)** Image analysis for co-localization of EdU and IL-2Rβ in a selected area of gill using Image J software. (a) A new merged image showing the PHA + rHis-IL-2 group in Figure 4A. Compared with the above image, magenta was represented IL-2Rβ-positive signals, aqua was represented EdU-positive signals, blue was represented EdU and IL-2Rβ double-positive signals, respectively. (b) Interactive 3D surface plot of the selected area (the position of the rectangle) of panel (a). (c) Analysis of co-localization of EdU and IL-2Rβ according to the optical density of three fluorescence in the selected area. Fifteen fish per group.

**FIGURE 6:**
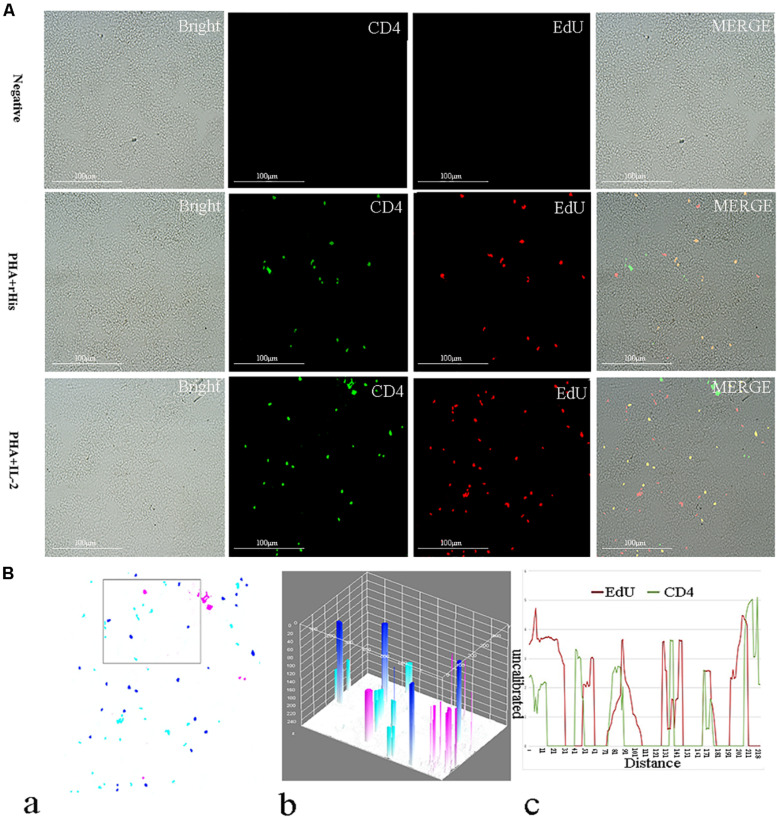
Co-localization analysis of EdU and CD4 in the spleen of flounder. **(A)** Indirect immunofluorescence of EdU and CD4 in flounder spleen after PHA + rHis, PHA + rHis-IL-2 injection on day 7; bar = 100μm. The unimmunized serum was used as primary antibody for negative control. **(B)** Image analysis for co-localization of EdU and CD4 in a selected area of spleen using Image J software. (a) A new merged image showing the PHA + rHis-IL-2 group in Figure 4A. Compared with the above image, magenta was represented CD4-positive signals, aqua was represented EdU-positive signals, blue was represented EdU and CD4 double-positive signals, respectively. (b) The interactive 3D surface plot of the selected area (the position of the rectangle) of (a). (c) Analysis of co-localization of EdU and CD4 according to the optical density of three fluorescence in the selected area (the position of the rectangle of a). 15 fish per group.

**FIGURE 7:**
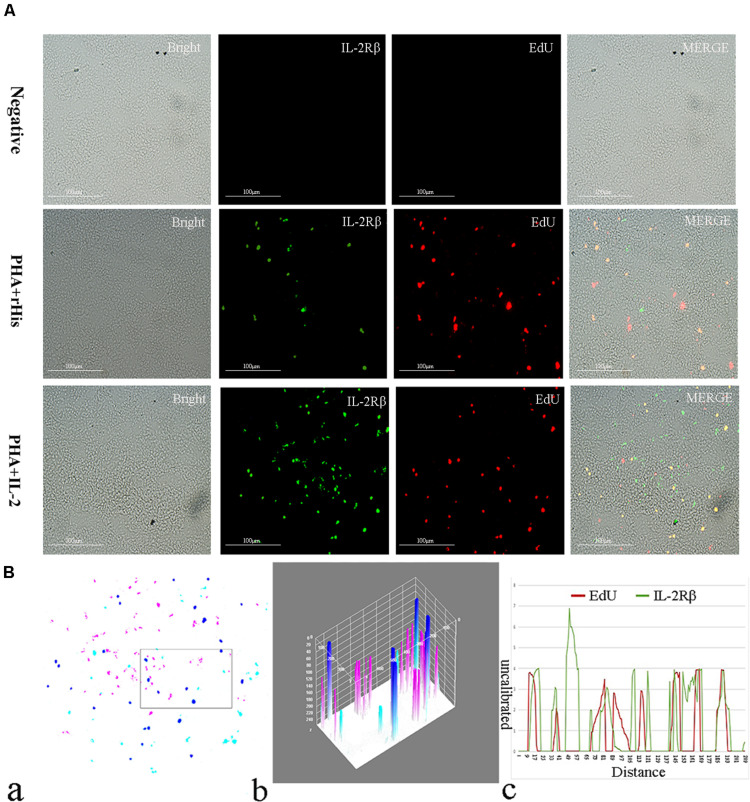
Co-localization analysis of EdU and IL-2Rβ in the spleen of flounder. **(A)** Indirect immunofluorescence of EdU and IL-2Rβ in flounder spleen after PHA + rHis, PHA + rHis-IL-2 injection on day 7; bar = 100μm. The unimmunized serum was used as primary antibody for negative control. **(B)** Image analysis for co-localization of EdU and IL-2Rβ in a selected area of spleen using Image J software. (a) A new merged image showing the PHA + rHis-IL-2 group in Figure 4A. Compared with the above image, magenta was represented IL-2Rβ-positive signals, aqua was represented EdU-positive signals, blue was represented EdU and IL-2Rβ double-positive signals, respectively. (b) Interactive 3D surface plot of the selected area (the position of the rectangle)of panel (a). (c) Analysis of co-localization of EdU and IL-2Rβ according to the optical density of three fluorescence in the selected area. Fifteen fish per group.

Cryostat sections and indirect immunofluorescence assay showed the CD4+ EdU+ and IL-2Rβ+ EdU+ signals in the gill and spleen of flounder after rfIL-2 injection. Fluorescence microscopy indicated a single positive of CD4+ or EdU+ signal. In addition, double positive signals (the co-location of EdU positive and CD4 positive signals, or IL-2Rβ positive and EdU positive signals) were also indicated, as both diffuse green fluorescence and red fluorescence corresponding to CD4+/EdU+ and IL-2Rβ+ EdU+ signals were also observed in the gill and spleen, respectively. The results showed that a considerable number of cells in the gill and spleen exhibited more double immunofluorescence staining in the rfIL-2 group than that in control group. The co-localization of EdU+ CD4+ cells and EdU+ IL-2Rβ+ cells indicated that rfIL-2 could enhance the proliferation of CD4+ and IL-2Rβ+ cells in gill (Figures 4A, 5A) and spleen (Figures 6A, 7A), respectively. By contrast, isotype controls with rabbit IgG and mouse IgG as control(non-related primary Abs) displayed no fluorescent signals. In addition, quantitative image analysis for co-localization of EdU and CD4, or EdU and IL-2Rβ, in the rfIL-2 group of the spleen and gill were analyzed using Image J software. An area of the epithelial layer in the new merged image was selected (black rectangle), and the interactive 3D surface plot of the selected area is shown in Figures 4–7b. The optical density of red and green fluorescence in the selected area was analyzed using Image J. The optical density was plotted and the overlapping pixels in the abscissa axis were considered to represent the area of co-localization of EdU+ and CD4+ cells, or EdU+ and IL-2Rβ+ (Figures 4–7c).

### CD4+ T Lymphocytes and IL-2Rβ+ Cell Were Increased After Treated With rfIL-2 *in vitro*

**FIGURE 8:**
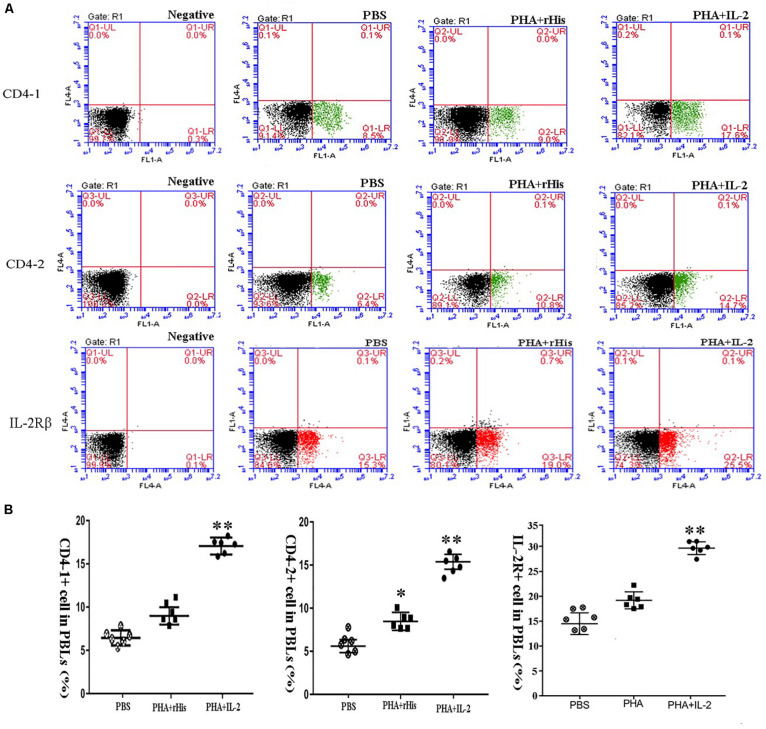
FCM assay investigating the proportion of CD4-1+ T lymphocytes, CD4-2+ T lymphocytes, and IL-2Rβ+ cells in peripheral blood of flounder cultured *in vitro* after rIL-2 addition at 72 h. **(A)** The representative dots of the results for CD4-1+ T lymphocytes, CD4-2+ T lymphocytes, and IL-2Rβ+ ells from PBS, PHA + rHis, and PHA + rHis-IL-2 groups at 72 h post addition. FCM was used to analyze the stained cells. **(B)** Percentages of CD4-1+ T lymphocytes, CD4-2+ T lymphocytes, and IL-2Rβ+ cells in different groups. All values are expressed as mean ± SD from the six of independent experiments. Asterisks (*) above the bar represent the statistical significant difference, *p < 0.05, **p < 0.01.

**FIGURE 9:**
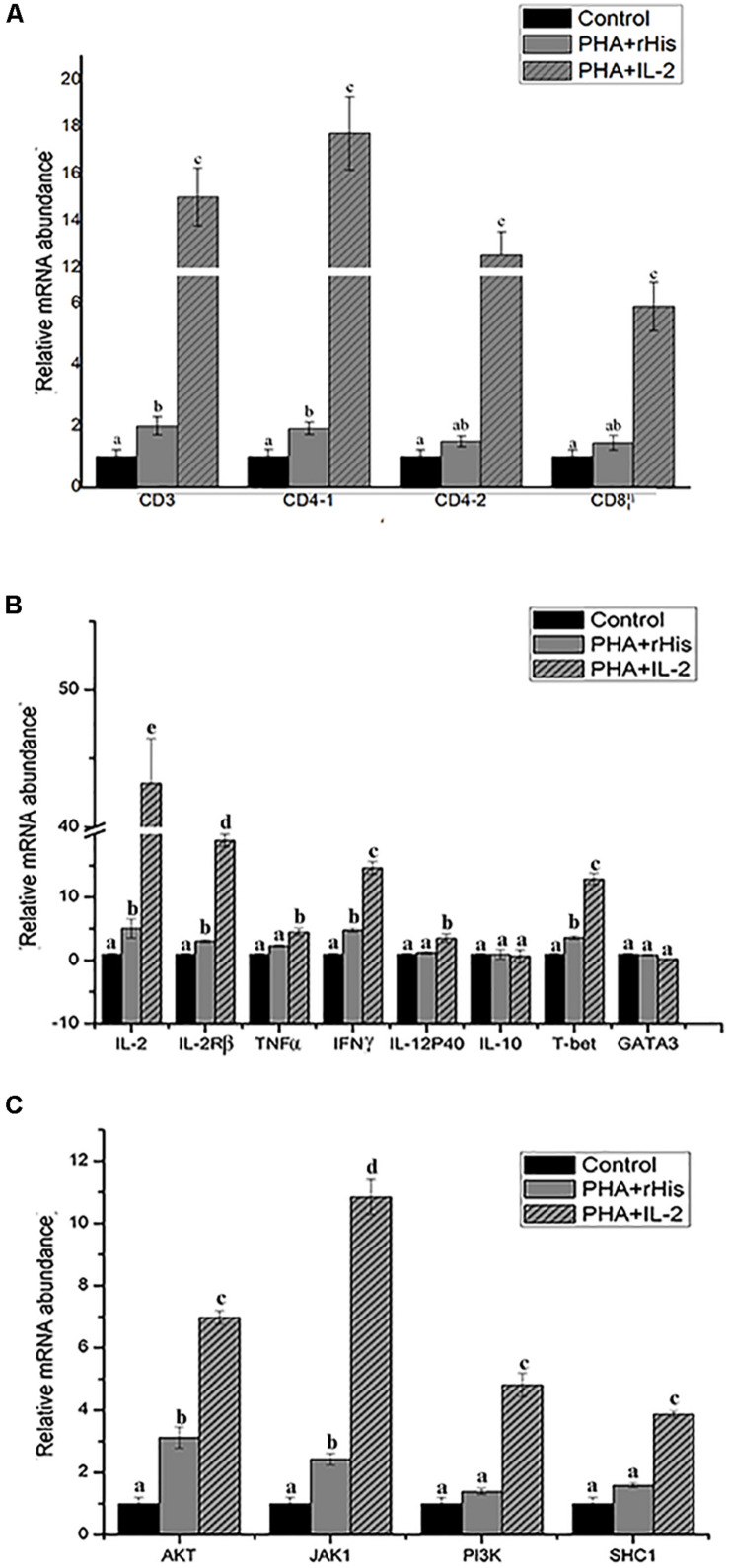
Relative gene expression in PBLs of flounder after addition of PBS, PHA + rHis, and PHA + rHis-IL-2 at 72 h. **(A)** The gene expression profile of T cell markers (CD3, CD4-1, CD4-2, and CD8β). **(B)** The gene expression profile of related cytokines (IL-2, IL-2Rβ, TNFα, IFNγ, IL-12p40, and IL-10), transcription factors (T-bet and GATA3). **(C)** The gene expression profile of IL-2 pathway (AKT, JAK1, PI3K, SHC1). The results represent the mean ± SD from the three independent experiments. Different letters above the bar represent the statistical significance (*p* < 0.05).

Flow cytometric analysis showed that the percentage of CD4-1+ T cells in the PBS group was 8.5 ± 1.04%, and as high as 9.0 ± 1.12%, 17.6 ± 1.94% in the PHA + His-tag protein and PHA + rfIL-2 groups, respectively. Similarly, the percentage of CD4-2+ T lymphocytes in the PBS and PHA + His-tag protein group was 6.4 ± 2.02% and 10.8 ± 1.87%, respectively, whereas that in the PHA + rfIL-2 group increased to 14.7 ± 2.12%. Furthermore, the percentage of IL-2Rβ+ cells increased to 25.5 ± 2.51% in the PHA+rfIL-2 group. The results demonstrated that CD4-1+ cells, CD4-2+ T lymphocytes, and IL-2Rβ+ cells showed significant increase (*p* < 0.05) when treated with rfIL-2 compared with the control groups (Figure 8). Quantitative-PCR analysis showed that the expression of T cell markers CD3, CD4-1, CD4-2, and CD8β also was upregulated significantly (*p* < 0.05) in PBLs after *in vitro* administration of frIL-2 (Figure 9A). Flow cytometry results are consistent with gene level results.

### Modulation of Th1-Related Cytokines and Transcription Factor Expression by rfIL-2 in PBLs

In order to analyze the modulation of the expression of genes stimulated by rfIL-2 in PBLs, the expression of genes encoding cytokines IL-2, IL-2Rβ, TNFα, IFNγ, IL-12p40, and IL-10, and those encoding the transcription factors T-bet and GATA3, was examined by Q-PCR after stimulation by rfIL-2 *in vitro* at 72 h. IL-2, IL-2Rβ, TNFα, IFNγ, and IL-12p40, which are critical cytokines involved in the Th1 pathway, were significantly upregulated by rfIL-2 (Figure 9B, *p* < 0.05). And, administration of rfIL-2 significantly upregulated the expression of T-bet (*p* < 0.05).

### RfIL-2 Upregulates the Expression of IL-2 Pathway Genes

In order to analyze the signaling pathway activated by IL-2, the expression of genes encoding AKT, JAK1, PI3K, and SHC1 was examined by Q-PCR. The results showed that, after 72h of stimulation by rfIL-2, the expression of these genes was significantly activated by rfIL-2 compared with that in control groups. In addition, JAK1 showed the highest level of upregulation of expression (Figure 9C).

### Anti-rfIL-2 and Anti-rfIL-2β Polyclonal Antibodies Decreasing the Percentage of T Cell Subsets

**FIGURE 10:**
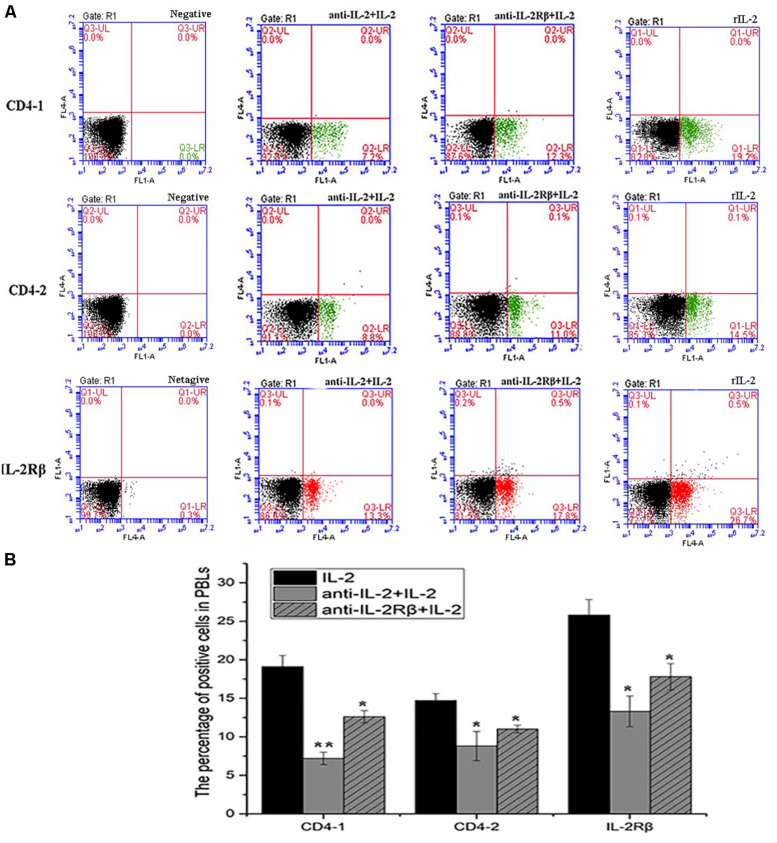
Blockade assay analysis of CD4-1+ T lymphocytes, CD4-2+ T lymphocytes, and IL-2Rβ+ cells in the peripheral blood of flounder by FCM. **(A)** The representative dots of show the results for CD4-1+ T lymphocytes, CD4-2+ T lymphocytes, and IL-2Rβ+ cells in anti-frIL-2 and anti-frIL-2β polyclonal antibody groups at 72h post addition. FCM was used to analyze the stained cells. **(B)** Percentages of CD4-1+ T lymphocytes, CD4-2+ T lymphocytes, and IL-2Rβ+ cells in different groups. All values are expressed as mean ± SD from the six of independent experiments.

To analyze the ability of anti-rfIL-2 and anti-rfIL-2β polyclonal antibodies to inhibit T cell number increase, anti-rfIL-2β polyclonal antibodies were diluted and incubated with PBLs. Similarly, anti-rfIL-2 polyclonal antibodies were pre-incubated with rfIL-2, and then incubated with PBLs. FCM results demonstrated that the percentages of CD4-1+ T lymphocytes were significantly decreased in all polyclonal antibody-blocked groups compared with that in the rfIL-2 group(19.2.0 ± 0.76%) when the same concentrations of mouse pre-immune serum and rfIL-2 were added (7.2 ± 0.93% and 12.6 ± 1.04%, respectively). Similarly, compared with that in the rfIL-2 group (14.5 ± 0.41%), the percentages of CD4-2+ T lymphocytes inhibited by polyclonal antibodies were 8.8 ± 1.41% and 11.0 ± 0.34%, respectively. Besides, compared with rfIL-2 group (26.8 ± 0.84%), the percentage of IL-2β+ cell was 13.3 ± 1.21% in anti-rfIL-2 polyclonal antibodies group, similarly, the percentage of IL-2β+ cell was17.8 ± 0.94% in anti-rfIL-2β polyclonal antibodies group, which were significantly decreased in the antibodies blocked groups (Figure 10).

**FIGURE 11:**
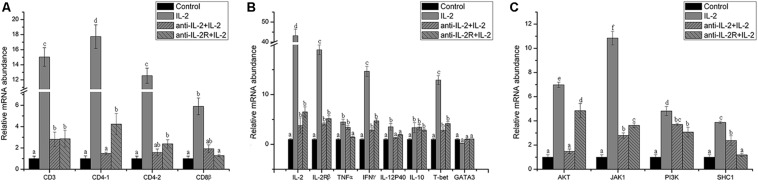
Relative gene expression in PBLs of flounder after blocking with anti-frIL-2 and anti-frIL-2β polyclonal antibodies at 72 h. **(A)** The gene expression profile of T cell markers (CD3, CD4-1, CD4-2, CD8β). **(B)** The gene expression profile of related cytokines (IL-2, IL-2Rβ, TNFα, IFNγ, IL-12p40, and IL-10), transcription factors (T-bet, GATA3). **(C)** The gene expression profile of IL-2 pathway (AKT, JAK1, PI3K, and SHC1). All data represent results obtained from at least three independent experiments. Different letters above the bar represent the statistical significance (*p* < 0.05).

In addition, the surface markers molecule expression of T lymphocytes was analyzed by Q-PCR. The upregulated expression of CD3, CD4-1, CD4-2, and CD8β also was inhibited significantly (*p* < 0.05) in PBLs after *in vitro* administration of anti-rfIL-2 and anti-rfIL-2β polyclonal antibody groups (Figure 11A). Similarly, compared with rIL-2 group, anti-rfIL-2 and anti-rfIL-2β polyclonal antibodies could inhibit the up-expression of those genes (Figure 11B). In anti-rfIL-2 and anti-rfIL-2β polyclonal antibody groups, the gene relative expression of AKT, JAK1, PI3K, and SHC1 were lower than that in rIL-2 group. And the relative expression of AKT in anti-rfIL-2 group and SHC1 in anti-rfIL-2β group showed no significant upregulation compared to control (Figure 11C). FCM and Q-PCR results showed that anti-rfIL-2 and anti-rfIL-2β polyclonal antibodies could inhibit T cell increase, respectively, and anti-rfIL-2 group showed a better inhibition effect than that of the anti-rfIL-2β group.

## Discussion

CD4+ T cells play an important role in the immune system. After antigen uptake, the macrophages first phagocytize the invading pathogen (26), release antigen information and submit it to CD4+ T cells. During this pivotal immunization process, the CD4+ T cells act as the “helpers and eyes” of the immune system (27–29).

In mammals, IL-2 is an important cytokine that regulates T cell proliferation (30). The proliferation of antigen-specific T cells during the immune response is critically dependent on the response to IL-2 (31, 32). The IL-2 gene of fish was cloned in Japanese pufferfish(*Fugu rubripes*)for the first time (33). After that, the IL-2 gene was successfully cloned in rainbow trout and large yellow croaker, and its biological activity was preliminarily analyzed (34, 35). In trout, the effect of rIL-2 on HK cells was analyzed, IL-2 could induce CD4 and CD8 genes upregulation in mRNA level (36). The results show that IL-2 could be used as an adjuvant to induce immune responses and improve the protective effect of candidate vaccines (19). The immunological characteristics and biological functions of IL-2, especially the effect of interaction with IL-2R on T lymphocytes, need to be further explored.

In order to analyze the interaction between IL-2 and IL-2Rβ and its promoting effect on the proliferation of CD4+ T lymphocytes in flounder, in this study, rfIL-2 was shown to induce the increase of PHA-activated T cell subpopulations after 72h of treatment *in vitro*. PHA as mitogens or Ags (the first signal of T lymphocyte activation), is mainly used to activate immune cells and lymphocytes, which synergy with cytokines to stimulate immune cell responses. Our previous studies showed that IL-2 could be used as a vaccine adjuvant (19), and it induces vaccine to produce high immune protection. IL-2 pretreatment can significantly improve the survival rates in flounder challenged with HIRRV (37). In addition, in our previous study, the results showed that without Ags activation, T cells cannot proliferate (21). And the biological function of IL-2 needs the activation of antigens. Compared with control groups (PHA control and PBS control groups), FCM results showed that rfIL-2 could increase the proportion of CD4-1+ and CD4-2+ T cell subpopulations of flounder. EdU as a thymidine nucleoside analog that could replace thymine (T) during the cell proliferation period and penetrate into the DNA molecule that is replicating (38). Cryostat sections and indirect immunofluorescence assay were used to analyze the CD4+/EdU+ and IL-2Rβ+/EdU+ cells in the gill and spleen of flounder. However, due to the limitation of magnification, single cells cannot be distinguished, nor can the nucleus and cell membrane be well distinguished. More positive signals were detected in the rfIL-2 injection group than that in the control group. Similar findings have reported that both trout IL-2 isoforms promoted PBL proliferation and sustained high level expression of CD4 and CD8, suggesting that trout IL-2 isoforms are T cell growth/survival factors mainly expressed by activated T cells (39). These studies in fish showed that a Th1-mediated mechanism regulated by IL-2 exists in bony fish, just like in mammals. It is shown that PHA is usually used as the antigen to activate T cells in the study of the effect of IL-2 on lymphocytes, similar study showed that human normal peripheral blood mononuclear cells are stimulated with PHA, the resulting T lymphoblasts can be propagated in IL-2 growth factor medium (40). In addition, the gene expression of CD3, CD4-1, CD4-2, and CD8β was also upregulated significantly (*p* < 0.05) in PBLs after *in vitro* administration of rfIL-2, which is consistent with the FCM data. Our previous studies showed that IL-2Rβ was expressed on CD4+ T lymphocytes (15). Combined with the data in this study, it’s speculated that the adjuvant effect of IL-2 is achieved by promoting the increase of T/B lymphocytes and antibody secretion, which enhancing the immunity of fish through the interaction with IL-2R (41, 42).

The pleiotropic effect of IL-2 on T, B, and NK cells is mediated by the IL-2 receptor which composed of α, β, and γ chain (43). These three subunits could be expressed individually or in different combinations to produce IL-2 binding receptors with significantly different affinities. Low-affinity intermediate receptors are first formed by β and γ chain subunits, and then co-expressed with α chain subunits to form high-affinity IL-2R (44). The α chain enhances IL-2R’s affinity for ligands and does not participate in signal transduction. However, both the β and γ chains belong to type I cytokine receptor superfamily, and they are responsible for signaling. The binding of IL-2 to the receptor complex can cause intracellular signal transduction events and eventually lead to cell proliferation. In this study, pull-down binding assays and ELISA demonstrated that His-rfIL-2 could bind specifically to GST-rfIL-2Rβ. In the blocked assay, anti-rfIL-2Rβ antibodies were used to block IL-2Rβ at the cell surface. Anti-rfIL-2Rβ antibody was incubated with PBLs at 22°C for 2h. Then, the unbound anti-rfIL-2Rβ antibody in the cell supernatant has been removed. And then, PHA+rfIL-2 was added to the cells. In addition, the percentage of IL-2Rβ+ cells in heath flounder PBLs was about 13–18% in our previous study. And after Keyhole limpet hemocyanin (KLH) injection, with the increase of T lymphocytes, the percentages of IL-2Rβ+ cells in PBLs were significantly increased (15). While in the blocking experiment, the IL-2Rβ on the cell surface are blocked by antibodies initially. Anti–rfIL-2Rβ antibody that bind to cell surface are also detected in FCM results. But the unbound antibody in the supernatant has been removed, it will not interfere with the result. Compared to the negative control group, the percentage of CD4-1+ and CD4-2+ T lymphocytes were inhibited by polyclonal antibodies. And the percentage of IL-2Rβ+ cells in anti-IL-2Rβ antibody group was lower than that in rfIL-2 group (25.5%, *p* < 0.05). Significant differences from the control group illustrate that rfIL-2–elicited biological function and immune response were downregulated by blocking with anti–rfIL-2Rβ and anti-rfIL-2 Abs. The data showed that modulation of IL-2 regulates CD4+ T lymphocyte increase via its interaction with IL-2Rβ. The similar research was that the IL-4 and IL-4Rα interaction in adaptive immunity of zebrafish (45). IL-2Rβ therefore might play an indispensable role in IL-2–elicited biological function.

The IL-2–IL-2R-mediated signaling pathway involves JAK-STAT, PI3K-AKT, and MAPK (46, 47). IL-2 signaling involves STAT5A and STAT5B activation via the Jak1 and Jak3 kinases, as well as signaling pathways dependent on GTPase Ras–mitogen-activated protein kinase and phosphatidylinositol-3-OH kinase9 (48). In the rainbow trout, key cytokines Th1 and Th2 cytokine are both upregulated in PBLs by IL-2A and IL-2B (39). The IL-2 signaling pathway-related genes, namely those encoding IL-2, IL-2Rβ, TNFα, IFNγ, IL-12p40, and IL-10, as well as those encoding transcription factors T-bet and GATA3, were detected by Q-PCR. RfIL-2 significantly upregulates the expression of IL-2, IL-2Rβ, TNFα, IFNγ, IL-12p40, and T-bet (*p* < 0.05), but downregulates that of IL-10 and GATA3 with no significant difference. This result shows that IL-2 acts as a Th1 cytokine that be involved in Th1 development in fish immunity. Further, the expression of AKT, JAK1, PI3K, and SHC1 was significantly upregulated by IL-2 compared with that in control groups. Among these, the expression of JAK1 showed the most significant upregulation. JAK1-STAT5 is the vital signal pathway of IL-2–IL-2R (49), and the genes of this pathway showed maximal induction in expression. In antibodies blocking experiment, compared with the negative control group, the genes was upregulated, IL-2Rβ as one of the receptors, its antibody could only specifically block IL-2Rβ on the cell surface, not all receptors. But compared with the rfIL-2 treatment group, the results of antibody blocking showed that the anti-IL-2Rβ could inhibit IL-2-elicited biological functions and immune responses, which plays an important role in this process. The results showed that the IL-2–IL-2R-mediated signaling pathway in flounder is similar to that in mammals.

In conclusion, this study showed that IL-2 interacts with IL-2Rβ and then increased the proportion of CD4+ T lymphocytes in flounder, and provided insights into the signaling pathway involved in IL-2 biological function. IL-2 is therefore crucial for the initiation of Th1-type immune responses and cell-related immunity in bony fish.

## Data Availability Statement

All datasets generated for this study are included in the article/supplementary material.

## Ethics Statement

All experimental methods were approved by the Instructional Animal Care and Use Committee of the Ocean University of China (permit number: 20180101).

## Author Contributions

XZ and JX contributed to the conception and design of this study, performed the experiments and statistical analysis, and drafted and revised the manuscript. XT and XS participated in the design of the study and helped analyze the experiments. HC assisted with reagent preparation and participated in data analysis. WZ edited the manuscript, and provided reagents and laboratory space. All authors read and approved this version of the final manuscript, and confirm the integrity of this work.

## Conflict of Interest

The authors declare that the research was conducted in the absence of any commercial or financial relationships that could be construed as a potential conflict of interest.
